# Interleukin‐6 positively correlates with cardiovascular disease predictor algorithms and biomarker in rheumatoid arthritis patients

**DOI:** 10.1111/jcmm.70028

**Published:** 2024-08-19

**Authors:** Seyed Askar Roghani, Afsaneh Shamsi, Cyrus Jalili, Farnaz Jalili, Ramin Lotfi, Nima Garman, Rezvan Rostampour, Mahdi Taghadosi

**Affiliations:** ^1^ Immunology Department, Faculty of Medicine Kermanshah University of Medical Sciences Kermanshah Iran; ^2^ Clinical Research Development Center, Imam Reza Hospital Kermanshah University of Medical Sciences Kermanshah Iran; ^3^ Medical Biology Research Center, Health Technology Institute Kermanshah University of Medical Sciences Kermanshah Iran; ^4^ University of Adelaide Adelaide South Australia Australia; ^5^ Blood Transfusion Research Center High Institute for Research and Education in Transfusion Medicine Tehran Iran; ^6^ Clinical Research Development Center, Tohid Hospital Kurdistan University of Medical Sciences Sanandaj Iran; ^7^ Department of Biology, Faculty of Basic Sciences University of Guilan Rasht Iran; ^8^ Cardiovascular Research Center, Health Technology Institute Kermanshah University of Medical Sciences Kermanshah Iran

**Keywords:** C‐reactive protein, cardiovascular disease, CXCR3, Interleukin‐6, rheumatoid arthritis

## Abstract

Chronic inflammation is believed as the main culprit of the link between cardiovascular disease (CVD) and rheumatoid arthritis (RA). Interleukin‐6 (IL‐6) is a pro‐inflammatory cytokine with a key role in RA pathophysiology and also correlates with joint destruction and disease activity. This study evaluates the association between IL‐6 plasma level and cardiac biomarker NT‐proBNP, HS‐CRP, CVD predictor algorithms, Framingham Risk Score (FRS) and Systematic Coronary Risk Evaluation (SCORE), as well as with CXCL9 and its receptor, CXCR3 in RA patients compared to the controls. Sixty RA patients (30 early and 30 late) and 30 healthy persons were included in this study. IL‐6 and NT‐proBNP plasma levels were measured by the ELISA. Also, HS‐CRP plasma levels were quantified using the immunoturbidimetric assay. The CVD risk was assessed by the FRS and SCORE. IL‐6 plasma levels were significantly higher in the early and late RA patients compared to the controls (*p* < 0.001). There was a positive correlation between IL‐6 with DAS‐28 (*p* = 0.007, *r* = 0.346), BPS (*p* = 0.002, *r* = 0.396), BPD (*p* = 0.046, *r* = 0.259), SCORE (*p* < 0.001, *r* = 0.472), and FRS (*p* < 0.001, *r* = 0.553), and a negative association with HDL (*p* = 0.037, *r* = −0.270), in the patients. Also, IL‐6 plasma level positively correlated with HS‐CRP (*p* = 0.021, *r* = 0.297) and NT‐proBNP (*p* = 0.045, *r* = 0.260) in the patients. Furthermore, a positive association was found between IL‐6 plasma levels and CXCL9 (*p* = 0.002, *r* = 0.386), and CXCR3 (*p* = 0.018, *r* = 0.304) in the patients. Given the interesting association between IL‐6 with various variables of CVD, IL‐6 may be considered a biomarker for assessing the risk for future cardiovascular events in RA patients.

## 1 INTRODUCTION

Rheumatoid arthritis (RA) is an inflammatory autoimmune rheumatic disease with unknown origin that primarily affects the small joints of the hands and feet, and also every tissue and organ of the body which ultimately causes the presence of many comorbidities, including cardiovascular disease (CVD).^1^, ^2^, ^3^ CVD in RA patients include a wide range of congestive and non‐congestive heart diseases, such as atherosclerosis, heart failure and peripheral vascular disease, and is considered the main reason for the 50% increased deaths in the RA population.^4^, ^5^, ^6^ The precise underlying cause connecting CVD and RA remains not fully understood. Still, according to the evidence, chronic systemic inflammation is proposed as the main culprit of the link between CVD and RA.^7^, ^8^, ^9^ The state of chronic inflammation in RA can also cause extra‐articular manifestations, like rheumatoid nodules and vasculitis, as well as cardiovascular, neurological, pulmonary, renal, gastrointestinal and hematologic diseases.^5^ In patients with RA, markers of active inflammation, like C‐reactive protein (CRP) levels, and the severity of the disease activity, are associated with cardiovascular risk, and the exacerbation of the inflammatory state in RA contributes to increasing CVD risk.^10^

Pro‐inflammatory cytokines, like tumour necrosis factor‐α (TNF‐α), interleukin‐1β (IL‐1β) and IL‐6, play a critical role in both CVD and RA pathogenesis. In the inflammatory model of RA, higher levels of pro‐inflammatory cytokines can affect both the vascular endothelium and traditional cardiovascular risk factors, which can lead to an increased risk of developing a variety of cardiovascular disorders in RA patients.^10^, ^11^ IL‐6—a pro‐inflammatory cytokine with a key role in RA pathophysiology—is significantly increased in both the serum and synovial fluid of RA patients and correlates with joint destruction and disease activity.^12^ The heightened IL‐6 expression is strongly related to developing a wide range of cardiovascular disorders, including atherosclerosis, myocardial infarction, heart disease and ischemic stroke. By stimulating the synthesis of acute phase reactants especially CRP, coagulation cascade, endothelial dysfunction, perpetuation of inflammatory responses and effects on lipid metabolism, IL‐6 can lead to the development and acceleration of CVD occurrence.^13^, ^14^, ^15^ In high‐risk patients, IL‐6 can predict long‐term CV events and mortality in symptomatic patients with stable coronary disease and a high risk of cardiovascular events.^14^, ^16^ The European League Against Rheumatism (EULAR) has recently recommended the need for predicting and managing cardiovascular disorders in RA patients.^17^ It has also emphasized improving and adjusting models and algorithms developed to predict the risk of cardiovascular disorders in the general population, like Systematic Coronary Risk Evaluation (SCORE) and Framingham Risk Score (FRS) for use in these patients.^4^, ^18^, ^19^ Given the role of IL‐6 in chronic inflammation and the established role of IL‐6 in both CVD and RA pathogenesis, this study aimed to assess the relationship between plasma levels of IL‐6 with a comprehensive profile of traditional cardiovascular risks, as well as the calculated scores of two CVD predictor algorithms, SCORE and FRS. To obtain a more accurate assessment of the likelihood of developing cardiovascular disorders, the correlation of IL‐6 plasma level with cardiac biomarker N‐terminal pro‐B‐type natriuretic peptide (NT‐proBNP), and high sensitivity‐CRP (HS‐CRP) was also evaluated in this study.

CRP, an acute‐phase protein synthesized by hepatocytes in response to IL‐6 stimulation, is believed to have a substantial role in inflammation progression.^20^ HS‐CRP is one of the most robust inflammatory biomarkers for predicting early incident CVD risk, and early elevation in HS‐CRP levels is linked to increased risk of developing CVD and all‐cause mortality.^21^, ^22^, ^23^ In addition, many previous studies have indicated that CRP is a marker of subclinical cardiovascular events in RA patients, and an increase in CRP levels is associated with an increased risk of cardiovascular disorders in this population.^24^, ^25^ NT‐proBNP is a biomarker that is commonly used as a significant indicator for diagnosing and predicting heart failure and cardiac dysfunction.^26^, ^27^ On the other hand, based on the evidence in patients with RA and cardiac insufficiency, the circulating level of NT‐proBNP also may be a prognostic biomarker.^28^ In a recent study, it was discovered a significant positive association between the cardiac biomarker NT‐proBNP and HS‐CRP with C‐X‐C motif ligand 9 (CXCL9) chemokine, a crucial inflammatory chemokine, which plays an essential role in the CVD and RA pathogenesis.^29^ Recently, CXCL9 was introduced as the most strong contributor to age‐related chronic inflammation (iAge) and insufficient cardiac and vascular performance.^30^ Furthermore, in a recent investigation, it was shown a remarkable association of this chemokine with various variable of CVD in RA patients.^31^ In the current study, for determining the possible prognostic role of IL‐6 in the development of cardiovascular events in the subclinical stages in RA patients, the association between plasma IL‐6 concentration and cardiac biomarker NT‐proBNP and HS‐CRP, CVD predictor algorithms, SCORE and FRS, as well as with CXCL9 and its receptor, CXCR3 was assessed.

## 2 MATERIALS AND METHODS

### 2.1 Study population

This cross‐sectional study included 30 early and 30 late rheumatoid arthritis (RA) patients referring to Imam Reza Hospital, Kermanshah University of Medical Sciences (KUMS) in the period between April 2022 and October 2022, as well as 30 healthy subjects. The diagnosis of RA was performed by expert rheumatologists based on the classification criteria of the American College of Rheumatology/The European League Against Rheumatism 2010 (ACR/EULAR 2010).^32^ Furthermore, 30 Kermanshah City‐resident healthy subjects who were matched to the RA patients in terms of age and sex and lacked any background of autoimmune and inflammatory disorders were selected as the control group. This study followed the Declaration of Helsinki and was conducted with approval from the Ethics Committee of the KUMS (Ethical code: IR.KUMS.MED.REC.1402.431) and all subjects signed a written informed consent before participation in the study.

### 2.2 Inclusion criteria

Patients diagnosed with rheumatoid arthritis (RA) only,^32^ without any other underlying diseases with personal consent. Early RA with onset of symptoms less than 24 weeks and not taking anti‐rheumatic drugs. Late RA patients with disease duration of 1 to 12 years who have been treated with anti‐rheumatic drugs for at least 1 year.

### 2.3 Exclusion criteria

Having a prior history of systemic rheumatic and autoimmune diseases other than RA, chronic liver and kidney diseases, endocrine, and metabolic disorders, such as type 1 and type 2 diabetes mellitus, hyper‐ or hypothyroidism, severe infection, cancer, CVD, or other forms of atherosclerosis‐associated disorders like stroke/transient ischemic attack, as well as pregnant women. Besides, patients or controls being treated with anti‐lipid drugs, like statins, were excluded from this study.

### 2.4 Isolation of plasma sample

Five millilitres of peripheral blood were collected from the participants on the same day of the clinical assessment. Plasma was instantly separated by centrifugation for 10 min at 3000× g and stored at −80°C until analysis.

### 2.5 Enzyme‐linked immunosorbent assay

The plasma levels of IL‐6 (Demeditec, Germany) (LOT 2109–3335) and NT‐proBNP (ZellBio GmbH, Germany) (Cat.NO: ZB‐11239C‐H9648) (assay range: 12.5–400 Pg/mL) were measured using Enzyme‐Linked Immunosorbent Assay based on the protocols of the ELISA kit.

### 2.6 Immunoturbidimetric assay

The concentration of high sensitivity‐CRP (HS‐CRP) in plasma samples was assessed using ADVIA 1800 Clinical Chemistry System (Siemens, Germany) based on latex‐enhanced immunoturbidimetric according to the manufacturer's instructions (assay range: 0.16–10 mg/L).

### 2.7 Assessment of fasting blood sugar and lipid profile

After 12 h of fasting and plasma separation, fasting blood sugar (FBS) was determined by glucose oxidase‐peroxidase method (Biosystems, Barcelona and Spain), and lipid profile including total cholesterol, high‐density lipoprotein cholesterol (HDL‐Col), low‐density lipoprotein cholesterol (LDL‐Col) and triglyceride was measured via enzymatic reactions using commercial kits according to manufacturer's instruction (Biosystem, Barcelona Spain) and results were read using fully automated 7020 chemistry analyser (Hitachi, Tokyo, Japan).

### 2.8 Disease activity score‐28 (DAS‐28) and body mass index (BMI) calculation

DAS‐28 was calculated by expert rheumatologists using the formula DAS28‐ESR = 0.56 (TJ)^½^ + 0.28 (SJ)^½^ + 0.70 ln (ESR) + 0.014 GH (TJ: number of tender joints from 28 joints, SJ: number of swollen joints from 28 joints, GH: global health, ESR: erythrocyte sedimentation rate), as formerly described by Prevoo et al.^33^ BMI was also measured for each participant using the formula weight in kilograms divided by the height in meters squared (kg/m^2^).

### 2.9 SCORE and FRS calculation

CVD risks were calculated using the CVD risk prediction algorithms, FRS and SCORE. Most of these calculators include conventional risk factors such as age, sex, blood pressure, smoking and plasma lipid levels. The SCORE was created and approved in 2003 from 12 European cohorts^18^ and FRS was derived and internally ratified in the USA cohort, to estimate the 10‐year risk of CVD.^34^, ^35^, ^36^ The SCORE and FRS were calculated by using valid online websites (http://www.heartscore.escardio.org/ and https://www.framinghamheartstudy.org/fhs‐risk‐functions/cardiovascular‐disease‐10‐year‐risk/, respectively).

### 2.10 Statistical analysis

Data analysis was performed using SPSS software version 24.0 (SPSS, Chicago, IL, USA) and by the software GraphPad Prisms® 6.0 (GraphPad Software, La Jolla, California, USA). The distribution of data normality was done by the 1‐sample Kolmogorov–Smirnov (K–S) test. The one‐way ANOVA test was used to compare data between three groups. The correlation analysis was also done using Spearman and Pearson's rank correlation. Results were presented as the mean ± standard error of the mean (SEM), and significance level was considered at *p* < 0.05.

## 3 RESULTS

### 3.1 Participants characteristic

The present study examined 30 early and 30 late RA patients, as well as 30 age‐ and gender‐matched healthy controls. There existed no significant difference between the studied participants in terms of age and sex. Moreover, therapy with conventional DMARDs significantly diminished the tender joint count (TJC), swollen joint count (SJC) and DAS‐28 relative to the early RA patients (*p* < 0.0001, *p* < 0.0001 and *p* < 0.001, respectively). Demographic information and dosage of disease‐modifying anti‐rheumatic drugs (DMARDs) in the studied groups are shown in Table 1.

**TABLE 1:** The DMARD dosage and the demographic information of the studied groups.

Variables	Early RA	Late RA	Control	*p*‐value
Number	30	30	30	
Age (years)	48.80 ± 13.01	49.67 ± 10.51	48.10 ± 12.07	
Sex	Male (*n* = 5) Female (*n* = 25)	Male (*n* = 5) Female (*n* = 25)	Male (*n* = 5) Female (*n* = 25)	
TJC	3.33 ± 3.50	0.23 ± 0.97		<0.0001
SJC	3.20 ± 3.46	0.23 ± 0.97		<0.0001
DAS‐28	3.60 ± 1.16	2.33 ± 0.66		<0.001
Smoking status	Positive (6.7%) (*n* = 2) Negative (93.3%) (*n* = 28)	Positive (10%) (*n* = 3) Negative (90%) (*n* = 27)	Positive (*n* = 0) Negative (*n* = 30)	
Medication				
MTX^1^ (%)	0	100	0	
HCQ^2^ (%)	0	100	0	
PSL^3^ (%)	0	100	0	

*Note*: Data are expressed as the Mean ± SEM; 1‐Methotrexate (7.5–25 mg per week), 2‐Hydroxychloroquin (200 mg per day), 3‐Prednisolone (5–10 mg per day).

Abbreviations: SJC, swollen joint count; TJC, tender joint count.

### 3.2 Comparing the BMI, BP, FRS, SCORE and plasma levels of FBS, lipid profile (HDL‐Col, LDL‐Col, TG, TC), IL‐6, NT‐proBNP, CXCL9, and HS‐CRP in patients (early + late) and control group

**FIGURE 1:**
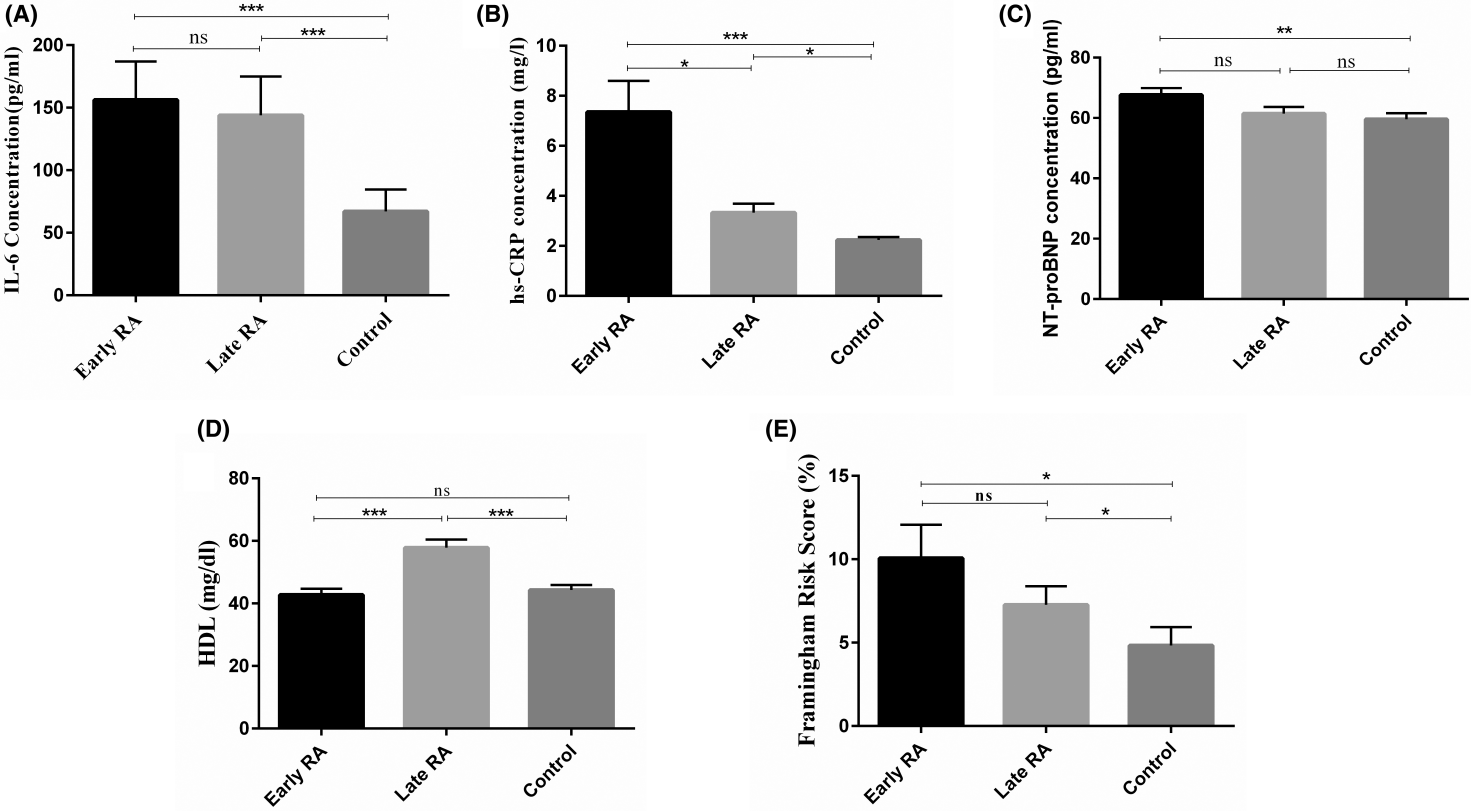
Comparing the plasma levels of IL‐6, HS‐CRP, NT‐proBNP, HDL, and FRS between three groups. The plasma level of IL‐6 and NT‐proBNP was quantified by the sandwich ELISA technique. HDL and HS‐CRP plasma levels also were measured by enzymatic reactions using commercial kits, which were read using a fully automated 7020 chemistry analyser and immunoturbidimetric assay, respectively. CVD risk was measured by the FRS calculator. (A) Comparing the plasma level of IL‐6 between study groups which significantly was higher in early and late RA patients compared to the control group (*p* < 0.001 and *p* < 0.001, respectively). (B) HS‐CRP was remarkably higher in early and late RA patients compared to the control group (*p* < 0.001 and *p* < 0.05) and was higher in the early RA group compared to the late RA group (*p* < 0.05). (C) NT‐proBNP plasma level was significantly higher in early RA compared to the control group (*p* < 0.01). (D) HDL was remarkably higher in late RA compared to early RA and control groups (*p* < 0.001 and *p* < 0.001, in order). (E) The SCORE obtained from the FRS algorithm was significantly higher in early and late RA patients compared to the control group (*p* < 0.05).

The mean BMI, BP, FRS, SCORE and plasma concentrations of FBS, lipids, HS‐CRP, NT‐proBNP, CXCL9 and IL‐6 in early RA, late RA and control groups are represented in Table 2. There was no statistically significant difference between the studied groups in terms of BMI, BPS, BPD, SCORE, FBS, LDL‐Col, TG and TC (*p* > 0.05). The plasma concentrations of IL‐6 were significantly increased in the early and late RA patients compared to the controls (*p* < 0.001) (Figure 1A). Moreover, HS‐CRP plasma levels were strikingly greater in the early and late RA patients relative to the controls and in the early RA relative to late RA patients (*p* < 0.001, *p* < 0.05 and *p* < 0.05, respectively) (Figure 1B). In the case of NT‐proBNP, its plasma concentrations were significantly enhanced in the early RA patients than in controls (*p* < 0.01) (Figure 1C). There was a significant increase in the HDL‐Col plasma levels following DMARDs therapy in under‐treatment RA patients, in comparison to the early RA patients (*p* < 0.001). HDL‐Col plasma levels were also meaningfully higher in late RA patients relative to the controls (*p* < 0.001) (Figure 1D). Besides, FRS significantly increased in the early and late RA patients compared to the controls (*p* < 0.05) (Figure 1E). Also, the plasma levels of CXCL9 were significantly higher in the early and late RA patients relative to the controls, as well as in the early RA relative to late RA patients (*p* < 0.0001, *p* < 0.001 and *p* < 0.001, in order) (Table 2).

**TABLE 2:** The mean BMI, BP and plasma levels of FBS, lipids, HS‐CRP, NT‐proBNP and IL‐6.

Variables	Reference value	Early RA (*n* = 30)	Late RA (*n* = 30)	Control (*n* = 30)	*p*‐value
BMI (kg/m^2^)		26.78 ± 5.01	24.76 ± 4.62	25.89 ± 3.69	0.223
BPS (mm Hg)	90–150	118.33 ± 20.52	115.33 ± 13.57	114 ± 15.44	0.593
BPD (mm Hg)	70–90	78 ± 7.14	80.66 ± 6.91	81 ± 4.02	0.191
FBS (mg/dL)	75–110	95.33 ± 16.08	91.44 ± 23.32	98.96 ± 17.37	0.093
HDL‐Col (mg/dL)	30–80	42.73 ± 10.67	57.86 ± 13.97	44.30 ± 8.82	<0.001
LDL‐Col (mg/dL)	Below 100	93.03 ± 19.54	102.90 ± 22.76	92.03 ± 22.43	0.105
TG (mg/dL)	Up to 200	131.46 ± 59.19	112.60 ± 41.18	117.06 ± 29.13	0.241
Cholesterol (mg/dL)	Up to 220	169.96 ± 31.41	176.36 ± 39.77	165.36 ± 25.03	0.427
FRS		9.96 ± 11.02	7.20 ± 6.16	4.71 ± 6.07	0.029
SCORE		11.53 ± 12.19	8.83 ± 6.77	6.13 ± 6.60	0.078
NT‐proBNP (pg/mL)	18.6–68.8	67.61 ± 12.47	61.43 ± 11.99	59.60 ± 10.69	0.016
HS‐CRP (mg/L)	1.1–3.8	7.35 ± 6.82	3.33 ± 1.95	2.23 ± 0.62	<0.001
IL‐6	38–98	156.26 ± 30.6	143.91 ± 30.96	67.01 ± 17.49	<0.001
CXCL9 (ng/L)	32.2–130.9	149.27 ± 35.11	127.54 ± 30.26	112.08 ± 21.66	<0.001

*Note:* Data are expressed as the Mean ± SEM.

Abbreviations: BMI, body mass index; BPD, diastolic blood pressure; BPS, systolic blood pressure; FBS, fasting blood sugar; HDL‐Col, high‐density lipoprotein cholesterol; HS‐CRP, High sensitivity C‐reactive protein; IL‐6, Interleukin 6; LDL‐Col, low‐density lipoprotein cholesterol; NT‐proBNP, N‐terminal pro–B‐type natriuretic peptide; TG, triglyceride.

### 3.3 Assessing the correlation between IL‐6 with variables in patient groups (early + late)

**FIGURE 2:**
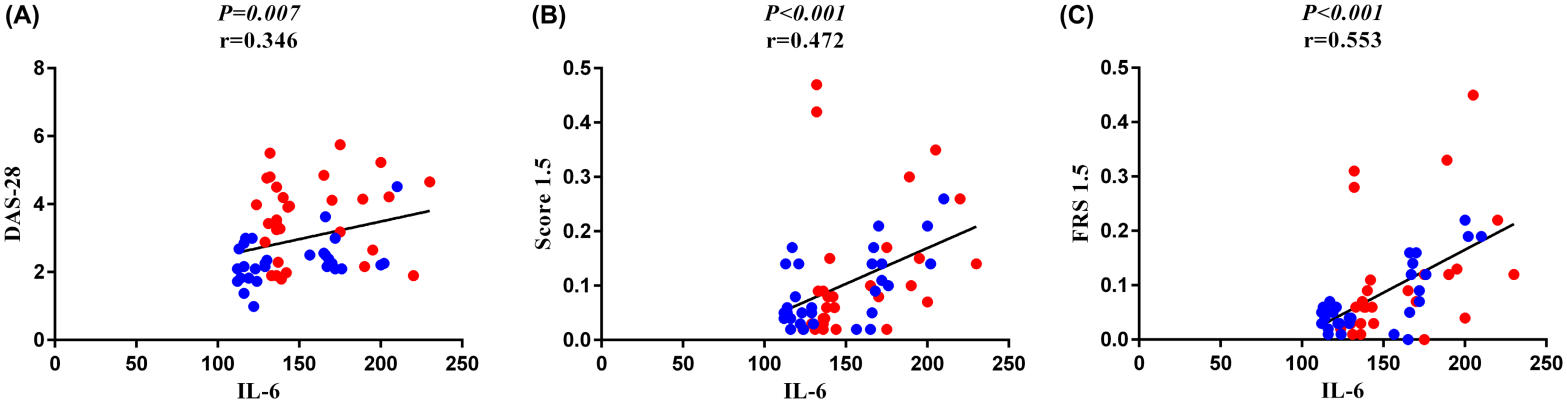
Association between IL‐6 plasma level with the DAS‐28, SCORE, and FRS. Correlation analysis was done using Spearman and Pearson correlations. (A) There was a significantly positive correlation between IL‐6 plasma level with DAS‐28 in patient groups (*p* = 0.007, *r* = 0.346). (B) There was a remarkably significant positive correlation between IL‐6 plasma level with SCORE in patient groups (*p* < 0.001, *r* = 0.472). (C) There was a significant positive correlation between IL‐6 plasma level with FRS in patient groups (*p* < 0.001, *r* = 0.553).

**FIGURE 3:**
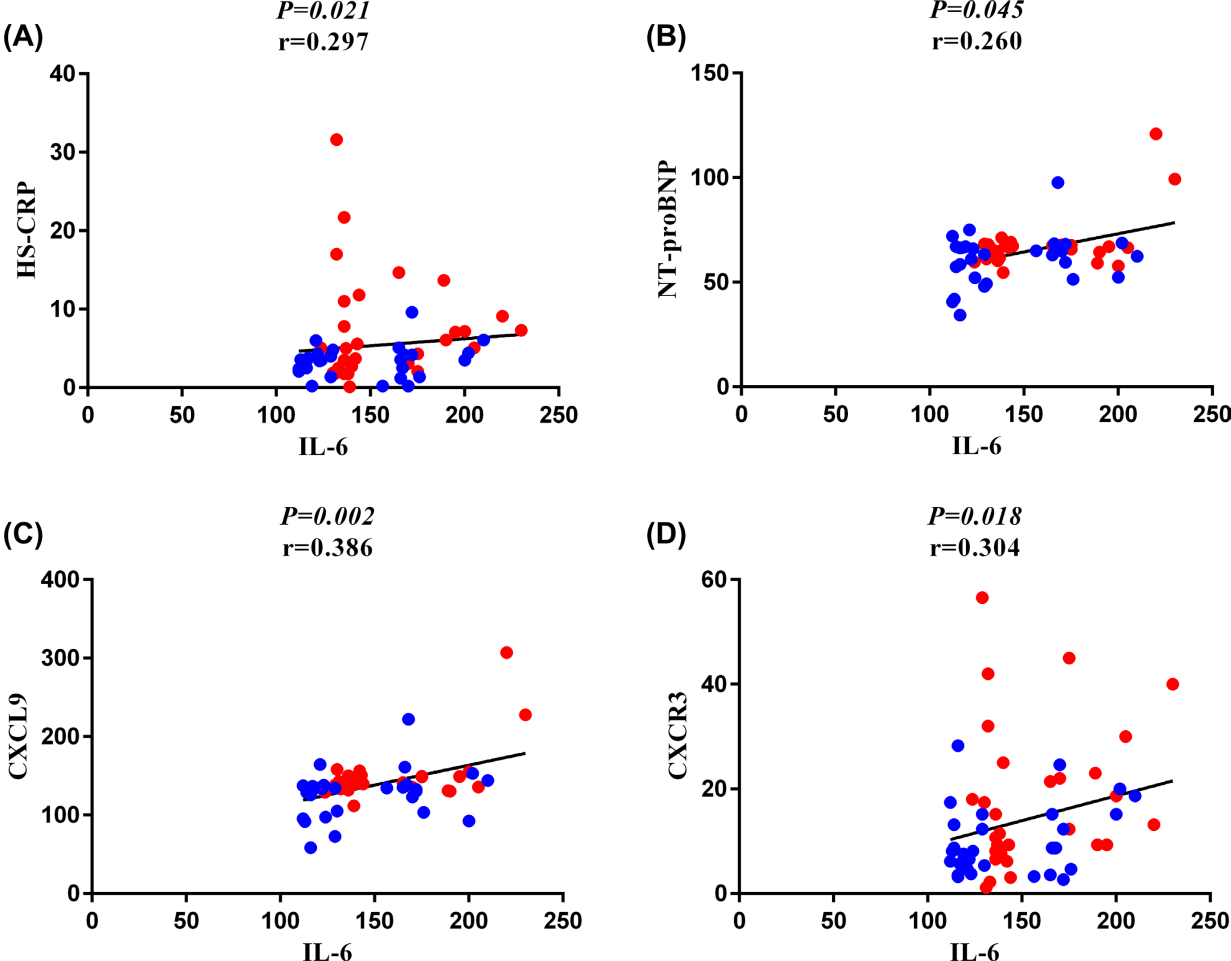
Association between IL‐6 plasma level with the HS‐CRP, NT‐proBNP, CXCL9 and CXCR3. (A) IL‐6 plasma level was positively correlated with HS‐CRP in RA patients (*p* = 0.021, *r* = 0.297). (B) IL‐6 plasma level was positively correlated with NT‐proBNP in RA patients (*p* = 0.045, *r* = 0.260). (C) There was a remarkable positive association between IL‐6 plasma level with CXCL9 in RA patients (*p* = 0.002, *r* = 0.386). (D) There was a significant positive correlation between IL‐6 plasma level with CXCR3 in RA patients (*p* = 0.018, *r* = 0.304).

There was a significantly positive correlation between IL‐6 with DAS‐28 (*p* = 0.007, *r* = 0.346), BPS (*p* = 0.002, *r* = 0.396), BPD (*p* = 0.046, *r* = 0.259), SCORE (*p* < 0.001, *r* = 0.472) and FRS (*p* < 0.001, *r* = 0.553), and a negative correlation with HDL‐Col (*p* = 0.037, *r* = −0.270), in early and late RA patients. Also, a significant positive association between the plasma level of IL‐6 with HS‐CRP (*p* = 0.021, *r* = 0.297), and NT‐proBNP (*p* = 0.045, *r* = 0.260) was found in early and late RA patients. Furthermore, a remarkable positive correlation between IL‐6 plasma levels with CXCL9 (*p* = 0.002, *r* = 0.386), and CXCR3 (*p* = 0.018, *r* = 0.304) was observed in early and late RA patients (Figures 2 and 3).

## 4 DISCUSSION

Chronic systemic inflammation is believed as the main culprit linking CVD and RA. However, finding an inflammatory marker that increases the risk of cardiovascular disorders in people with rheumatoid arthritis is an unmet need. In this study, for the first time, the correlation between the plasma concentration of IL‐6 with the most popular and validated CVD predictor algorithm, FRS and SCORE, and also with cardiac biomarker NT‐proBNP, and HS‐CRP was evaluated in early and late RA patients.^37^, ^38^

The plasma levels of IL‐6 were significantly higher in early and late RA patients compared with the healthy subjects. Furthermore, it was found that IL‐6 plasma levels correlated with RA activity parameters, DAS‐28. This is in accordance with the knowledge that in RA, IL‐6 is involved in various aspects of systemic inflammation and plays a pivotal role in the pathogenesis of joint destruction. It also can be considered as an indicator in evaluating the severity of disease activity and is associated with an increased risk of developing comorbidity conditions associated with RA, including cardiovascular events.^39^, ^40^ Given that IL‐6 plays a direct role in driving cardiovascular disease events related to chronic inflammation of RA, in the following, for the first time, the correlation between the plasma level of IL‐6 with components and scores obtained from CVD risk prediction algorithms, SCORE and FRS, and well established cardiac biomarker NT‐proBNP, and HS‐CRP was evaluated in RA patients.^41^ It was found a clear positive relationship between IL‐6 plasma levels and systolic and diastolic blood pressure. These results were in parallel with previous studies that showed systolic blood pressure and hypertension were positively correlated with IL‐6 plasma levels.^42^, ^43^ According to the documents, under the pathological condition of RA which is accompanied by an increased level of inflammatory mediators, IL‐6 can mediate thrombo‐inflammatory responses associated with angiotensin II, a critical determinant that is responsible for both thromboinflammation and hypertension, so targeting IL‐6 signalling pathway could help manage thrombo‐inflammatory diseases and hypertension.^43^

It is well known that the presentation of an atherogenic lipid profile, characterized by elevated total cholesterol (TC), total glyceride (TG) and low‐density lipoprotein cholesterol (LDL‐Col), as well as decreased high‐density lipoprotein cholesterol (HDL‐Col), can increase the risk of cardiovascular disease (CVD). However, in RA patients, there is a contradictory phenomenon called the “lipid paradox” attended with a decrease in TC and LDL‐Col.^44^, ^45^ Furthermore, HDL‐Col function was impaired and showed a pro‐inflammatory and proatherogenic character which loses its ability to remove cholesterol and increases the risk of CVD in RA patients.^46^ The present study showed a significant increase in plasma HDL‐Col levels in under‐treatment RA patients which may be due to DMARDs consumption documented in previous studies.^47^ Interestingly, the plasma HDL‐Col level showed a remarkable negative correlation with plasma levels of IL‐6 in these RA patients. According to the hypothesis that inflammation causes dyslipidemia in RA patients, the finding of this study can disclose the possible impact of IL‐6, as an important inflammatory agent, in dyslipidemia and increase the risk of CVD in RA patients.^45^, ^48^

To the best of our knowledge, in this study, it was shown a significant positive correlation between the plasma level of IL‐6 with the known CVD risk predictor algorithms, SCORE and FRS. IL‐6 has a crucial association with cardiovascular diseases, including atherosclerosis, hypertension, cardiac fibrosis and cardiomyopathy.^49^ IL‐6 also increases the risk of CVD through endothelial dysfunction by inducing CRP synthesis in the liver, which provokes leukocytes recruitment and perpetuates inflammatory responses.^13^ Using immunoturbidimetry assay, high‐sensitivity CRP (HS‐CRP) was evaluated in the plasma of RA and control groups and a significant positive correlation was observed between IL‐6 and HS‐CRP plasma levels in the patient groups. HS‐CRP diagnoses a low‐grade inflammation and is used as a potential biomarker for predicting cardiovascular disease risk, although IL‐6 also may have a prognostic role in subclinical cardiovascular events in high‐risk patients.^16^, ^50^ The existence of a clear positive association between IL‐6 and traditional CV risk factors, well‐established CVD predictor algorithms, FRS and SCORE, and pro‐inflammatory sensitive marker, HS‐CRP, in the present study, suggests that IL‐6 may play a prognostic role in the development of cardiovascular events in the subclinical stages. However, further investigations are needed to better understand this issue. In the following sections, we will examine more aspects of this issue.

NT‐proBNP is another potent predictor of CVD events which is secreted from cardiac myocytes in the early stages of cardiac diseases and used as a reliable serum biomarker for heart disorders screening and diagnosis even in asymptomatic stages.^51^, ^52^ In this study, the relationship between the plasma level of IL‐6 and NT‐proBNP was investigated in RA patients and a positive correlation was found between them. In parallel to our results, another study has shown that there is a connection between IL‐6 and NT‐proBNP plasma levels in patients who are suffering from acute heart failure. This study has also suggested that the combination of evaluations of IL‐6 and NT‐proBNP can be a valuable tool in predicting the prognosis of such patients.^53^ In RA patients, it has also been documented that the circulating level of NT‐proBNP can serve as a prognostic biomarker for the evaluation of cardiac disease.^28^

In a previous study, it was discovered a notable correlation between NT‐proBNP and CXCL9, an inflammatory chemokine that has a crucial role in the pathogenesis of rheumatoid arthritis (RA) and recently has also been introduced as a pivotal contributor to impaired cardiac and vascular function.^29^, ^30^ In the current study, the association between IL‐6 and CXCL9 was investigated in RA patients and interestingly a significant positive correlation was found between them. Furthermore, it was also discovered a remarkable positive correlation between IL‐6 and CXCR3, CXCL9 receptor. The pathways that are mediated by cytokines and chemokines have an undeniable role in the connection between cardiovascular disease events and RA. In this study, the presence of a strong association between IL‐6 and CXCL9/CXCR3 as two signatures of inflammatory cytokine and chemokine, respectively, in RA patients has further confirmed this notion. Noteworthy, the use of other drugs, like non‐steroidal anti‐inflammatory drugs (NSAIDs) and cyclooxygenase‐2 inhibitors (COX2i), by the studied RA patients (self‐medication) for declining their pain before RA diagnosis and the direct effects of these drugs on inflammatory conditions and disease‐involved factors, for instance IL‐6 and CXCL9/CXCR3, and our inability to exclude the possible effects of these drugs can be considered as a limitation of this study. Besides that, applying numerous exclusion criteria is another limitation of the study.

## 5 CONCLUSION

In conclusion, considering the existence of the interesting association between IL‐6 with various variables of CVD, SCORE and FRS, and screening cardiac biomarker in RA patients, IL‐6 may be considered a biomarker for the assessment of the risk for cardiovascular events in the subclinical stages of RA population.

## AUTHOR CONTRIBUTIONS

**Seyed Askar Roghani:** Data curation (equal); investigation (equal); validation (equal); writing – original draft (equal). **Afsaneh Shamsi:** Data curation (equal); investigation (equal); validation (equal); writing – original draft (equal). **Cyrus Jalili:** Methodology (equal); writing – review and editing (equal). **Farnaz Jalili:** Methodology (equal); writing – review and editing (equal). **Ramin Lotfi:** Data curation (equal); investigation (equal); writing – original draft (equal); writing – review and editing (equal). **Nima Garman:** Investigation (equal); writing – review and editing (equal). **Rezvan Rostampour:** Methodology (equal); writing – review and editing (equal). **Mahdi Taghadosi:** Conceptualization (equal); funding acquisition (equal); methodology (equal); project administration (equal); software (equal); supervision (equal); validation (equal); writing – review and editing (equal).

## FUNDING INFORMATION

This work was supported by the Kermanshah University of Medical Sciences grant number (4020751).

## CONFLICT OF INTEREST STATEMENT

The authors have no relevant financial or non‐financial interests to disclose.

## CONSENT TO PARTICIPATE

Informed consent was taken from all individual participants included in the study.

## CONSENT TO PUBLISH

The authors confirm that human research participants provided informed consent for the publication of the manuscript results.

## Data Availability

Data Availability The datasets generated during and/or analyzed during the present study are available from the corresponding authors upon reasonable request.
